# Factors Associated With Completion of Isotretinoin Therapy in Acne Patients Reporting Mood Disturbance

**DOI:** 10.1111/jocd.70106

**Published:** 2025-04-17

**Authors:** Joshua Farrell, Thomas Jonathan Stewart, Robert Rosen

**Affiliations:** ^1^ Southern Suburbs Dermatology Kogarah New South Wales Australia; ^2^ Department of Dermatology Addenbrookes Hospital Cambridge UK

**Keywords:** acne vulgaris, isotretinoin, mood disturbance

Dear Editor,

1

Isotretinoin is frequently discontinued in acne patients who report mood disturbance despite systematic reviews showing that it is not associated with an increased risk for depression and may even ameliorate depressive symptoms [1, 2]. The viability of completing isotretinoin therapy in acne patients reporting mood disturbance has not been fully explored. In this study, we characterize our cases of acne vulgaris treated with isotretinoin where mood disturbance was reported early in the course, comparing cases that completed with those that did not complete treatment.

We searched the electronic medical records at a large dermatology clinic for adults with acne vulgaris who commenced isotretinoin reporting mood disturbance. It was their first course of isotretinoin prescribed in doses ranging from 0.2 to 0.7 mg/kg. All patients were reviewed within 3 months of initiation. Completing the treatment course was defined as receiving a minimum cumulative dose of 150 mg/kg (facial and truncal disease) and 120 mg/kg (facial disease only). Patients with a prior history of mood disorder and/or reporting suicidal ideation were excluded. A chi‐squared test was used, and *p* values of less than 0.05 were considered statistically significant.

Five hundred and thirty‐two eligible cases were retrieved. One hundred and forty patients discontinued, whilst 392 continued the medication, with 128 remaining on their original dose and 264 on a lower dose. Two hundred and fifty‐three patients completed the full course. There were no episodes of self‐harm or suicide. Two hundred and ninety‐two patients were female, and the mean age was 21 years. Patient age 25 years or older (*p* = 0.002), significant therapeutic response in the first 3 months (*p* = 0.007), family history of acne vulgaris (*p* = 0.040), and formal psychiatric input (*p* < 0.001) were associated with an increased likelihood of completing the medication course (Table 1). Other significant associations were age ≥ 25 years with abnormal blood test results (*p* = 0.032) and female sex with other prescribed acne treatments (*p* = 0.112).

**TABLE 1:** Analysis of predictors of isotretinoin course completion.

	Did not complete course (*n* = 279)	Completed course (*n* = 253)	*p*
Age			
< 25 years	240	192	0.002
≥ 25 years	39	61	
Sex			
Male	120	120	0.306
Female	159	133	
Site/s affected			
Face	233	218	0.395
Trunk ± face	46	35	
Family history of severe acne vulgaris			
Yes	95	106	0.040
No	110	81	
Efficacy (assessed within 3 months)			
Nil to minimal	227	181	0.007
Significant	52	72	
Patient reported adverse effects (excluding xerosis)			
Yes	18	13	0.518
No	261	240	
Isotretinoin dose reduced			
Yes	145	119	0.492
No	75	53	
Other prescribed treatment for acne			
Yes	43	42	0.708
No	236	211	
Abnormal blood test/s results			
Yes	24	18	0.525
No	255	235	
Formal psychiatric input			
Yes	158	211	< 0.001
No	121	42	

Overall adherence to isotretinoin therapy in acne patients is around 80% [1]. Patients and prescribers are more highly motivated to continue on a medication that is providing clinical benefit, irrespective of its adverse effects [3]. Older patients perceive medication safety behaviors to be more important and more closely adhere to monitoring practices compared to younger patients [4]. Older patients may also have exhausted a relatively higher number of alternative treatments prior to isotretinoin.

Acne vulgaris is associated with an increased risk of developing depression independent of dermatologic therapy. The probability of developing major depressive disorder is 18.5% in patients with acne compared to 12% in the general population [5]. As such, patients may have had family members with acne not treated with isotretinoin who suffered concurrent mood changes, and other family members successfully completing courses of isotretinoin whilst experiencing mood disturbance may have reassured prescribers.

Patients who consulted a psychiatrist were more likely to have received specific psychiatric treatment, possibly resulting in better control of their mood symptoms. The prescribing dermatologist might also have gained additional confidence in continuing the isotretinoin in these patients, knowing psychological support was readily available.

An association was not found between a reduction in dose and completing the course of isotretinoin, suggesting that where cessation was attributed to the medication's adverse effects, it was not dose‐related. Historically, any relationship that may exist between isotretinoin and psychiatric adverse effects has been considered idiosyncratic.

Abnormal liver function and cholesterol test results did not increase the likelihood of the medication being discontinued. It has been shown that isotretinoin cessation is not necessary in most cases, as the biochemical abnormalities are usually mild, reversible, and occur in patients who do not possess other metabolic risk factors [6].

In summary, our findings suggest that isotretinoin therapy is successfully completed in the majority of acne patients who report mood disturbance in the early stages of the course. Older age, a family history of acne vulgaris, favorable therapeutic results early in the course, and formal psychiatric input may increase the likelihood of course completion in this cohort.

## Author Contributions

All authors contributed equally to this project.

## Conflicts of Interest

The authors declare no conflicts of interest.

## Data Availability

The data that support the findings of this study are available on request from the corresponding author. The data are not publicly available due to privacy or ethical restrictions.
